# Digital Cognitive Behavioural Therapy for Insomnia Delivered Within a Crenotherapy Setting: Results from a Multicentre Proof-of-Concept Randomised Controlled Trial

**DOI:** 10.3390/jcm15062176

**Published:** 2026-03-12

**Authors:** Julie Lenoir, Marie Mengarduque, Julien Coelho, Pierre-Alexis Geoffroy, Émilie Denéchère, Bruno Aouizerate, Nematollah Jaafari, Pierre Philip, Jacques Taillard, Olivier Dubois, Jean-Arthur Micoulaud-Franchi

**Affiliations:** 1 Health College, University of Bordeaux, F-33000 Bordeaux, France; marie.mengarduque@gmail.com (M.M.); julien_coelho78@hotmail.fr (J.C.); emilie.denechere03@gmail.com (É.D.); pr.philip@free.fr (P.P.); jacques.taillard@u-bordeaux.fr (J.T.); jarthur.micoulaud@gmail.com (J.-A.M.-F.); 2 Laboratoire SANPSY, CNRS UMR 6033, CHU Pellegrin, Tripode 13ème étage, aile 3, Place Amélie-Raba-Léon, F-33076 Bordeaux, France; 3 Service Universitaire de Médecine du Sommeil, CHU de Bordeaux, Place Amélie Raba-Léon, F-33000 Bordeaux, France; 4 Département de Psychiatrie et D’addictologie, AP HP, GHU Paris Nord, DMU Neurosciences, Hôpital Bichat Claude Bernard, F-75018 Paris, France; pierre.a.geoffroy@gmail.com; 5 NeuroDiderot, Inserm U1141, Université de Paris, F-75019 Paris, France; 6 Centre de Référence Régional des Pathologies Anxieuses et de la Dépression, Pôle de Psychiatre Générale et Universitaire, Centre Hospitalier Charles Perrens, F-33000 Bordeaux, France; bruno.aouizerate@u-bordeaux.fr; 7 Laboratoire NutriNeuro (UMR INRA 1286), Université de Bordeaux, F-33000 Bordeaux, France; 8 Centre Hospitalier Henri Laborit, F-86000 Poitiers, France; nemat.jaafari@ch-poitiers.fr; 9 Centre de Recherches sur la Cognition et l’Apprentissage, F-86000 Poitiers, France; 10 Clinique de Saujon et Établissement Thermal, BP 30, F-17600 Saujon, France; odubois@thermes-saujon.fr

**Keywords:** anxiety, crenotherapy, digital cognitive behavioural therapy, insomnia, randomised controlled trial, sleep behaviour

## Abstract

**Background/Objectives**: Insomnia disorder is highly prevalent and disabling, yet access to cognitive behavioural therapy for insomnia (CBT-I), the recommended first-line treatment, remains limited. Digital CBT-I (dCBT-I) offers scalable alternative; however, treatment outcomes vary according to intervention format and delivery context. This study evaluated whether delivering dCBT-I within a structured, medically supervised crenotherapy context improved insomnia symptom severity compared with stand-alone dCBT-I. **Methods**: In this multicentre proof-of-concept randomised controlled trial, 66 adults with insomnia disorder were allocated to receive either stand-alone dCBT-I (*n* = 38) or dCBT-I delivered within a 3-week standardised crenotherapy programme (medically supervised thermal spa treatment; *n* = 28). The primary outcome was change in Insomnia Severity Index (ISI) scores from pre- to post-treatment. Secondary outcomes included subjective sleep parameters (e.g., sleep efficiency and sleep onset latency), sleep-related functioning, pre-sleep arousal, anxiety and depressive symptoms. Engagement and satisfaction were assessed as additional descriptive outcomes. **Results**: Both groups showed significant improvements in insomnia severity, sleep parameters, and psychological symptoms. However, the primary between-group comparison did not demonstrate a statistically significant additive effect of crenotherapy on insomnia severity. ISI outcomes did not differ between the crenotherapy-delivered and stand-alone dCBT-I groups. Nevertheless, post hoc exploratory subgroup analyses suggested that, among participants younger than 60, delivery of dCBT-I within a crenotherapy care setting was associated with greater improvements in insomnia symptoms compared with stand-alone dCBT-I (mean ISI change: 10.4 vs. 5.4, *p* = 0.030). In a separate subgroup analysis, among participants with baseline anxiety symptoms, dCBT-I delivered within a crenotherapy care setting was associated with a greater reduction in anxiety compared with stand-alone dCBT-I (*p* = 0.030). Engagement and satisfaction were high in both groups, with no significant differences. **Conclusions**: Delivering dCBT-I within a crenotherapy context appears feasible and may offer specific benefits for specific subpopulations, particularly younger individuals and those with comorbid anxiety. These findings support further investigation of context-sensitive digital models to improve personalisation and accessibility of insomnia treatment.

## 1. Introduction

Insomnia disorder, affecting approximately 15.8% to 19% of the population [1] is characterised by persistent difficulties initiating or maintaining sleep, accompanied by daytime impairments such as fatigue, low energy, mood disturbances, and cognitive complaints [2]. Beyond its impact on daily functioning, insomnia disorder is also a well-established risk factor for anxiety and depressive disorders [3,4]. A key mechanism underlying the maintenance of insomnia disorder is classical conditioning, whereby the bed and bedroom become associated with arousal and anxiety following an acute stressor [5,6]. This can perpetuate sleep disturbances even after the initial stressor has resolved [7]. Cognitive processes such as worry and rumination further sustain hyperarousal and the development of insomnia disorder [8,9,10].

Current clinical guidelines recommend Cognitive Behavioural Therapy for Insomnia disorder (CBT-I) as the first-line treatment, given its more durable effects and fewer adverse effects compared with pharmacological approaches [11,12,13,14]. However, access to CBT-I is limited by the availability of trained clinicians, costs, and geographical disparities [15,16]. To address this, digital CBT-I has emerged as a scalable alternative [17]. Nevertheless, the literature reports heterogeneous results regarding the efficacy of dCBT-I. While several studies report outcomes comparable to those of face-to-face CBT-I (FtF CBT-I), others underscore substantial variability in treatment effectiveness as a function of patient characteristics, intervention modalities (automated-vs. guided CBT-I), and contextual factors (internet-vs. group-delivered CBT-I) [18,19,20].

Although effective, dCBT-I outcomes vary according to mode of delivery and the care context in which the intervention is implemented. dCBT-I encompasses heterogeneous delivery models, ranging from therapist-guided, web-based programs to automated interventions incorporating algorithm-driven or virtual therapeutic feedback, and fully automated unguided interventions [21]. A network meta-analysis by [22] showed that web-based dCBT-I incorporating automated therapeutic feedback achieved outcomes comparable to therapist-guided web-based dCBT-I, whereas fully automated interventions without guidance were associated with lower effectiveness. A meta-analysis by [17] demonstrated that dCBT-I was non-inferior to face-to-face CBT-I within a pre-specified non-inferiority margin on the Insomnia Severity Index, indicating that digital delivery per se does not imply reduced clinical effectiveness.

Beyond intervention format, the broader context of treatment delivery appears to shape how dCBT-I is accessed and implemented. Although lower levels of therapist involvement in some digital formats have been associated with reduced engagement and higher dropout rates [23,24,25], evidence indicates that the relationship between engagement, adherence, and treatment outcomes is complex and inconsistent. Completion rates do not reliably predict clinical outcomes [25], and systematic review evidence reports few and inconsistent associations between adherence measures and CBT-I outcomes [26]. Engagement challenges are also observed in therapist-delivered CBT-I, and treatment dose or session number does not appear to be a primary determinant of effectiveness [27].

**Figure 1:**
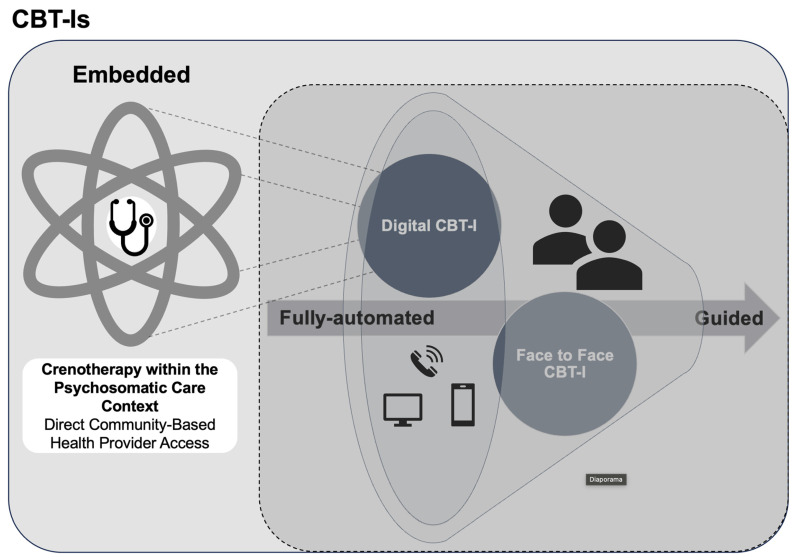
Integrated Care Context for Scalable Delivery of dCBT-I Within Crenotherapy Psychosomatic Treatment Framework. Adapted from [21,32].

At a population level, these issues must be interpreted within the broader context of a substantial treatment gap in insomnia care, with many individuals reporting insomnia symptoms but few receiving evidence-based treatment [28]. Access to digital health interventions also remains uneven due to persistent socioeconomic and structural barriers [29,30]. In addition, many patients with chronic insomnia disorder are poor candidates for pharmacological treatments due to physical dependence, age, comorbidities, pregnancy-related concerns, or polypharmacy, reinforcing the relevance of non-pharmacological and scalable interventions such as dCBT-I [14]. In some cases, “combining medication with CBT-I may provide an added benefit during the initial course of therapy, but the clinical significance of such added benefit is unclear” in the long term [31]. Together, this evidence highlights the importance of evaluating dCBT-I within a broader supportive care context that may facilitate access and delivery, rather than focusing solely on intervention format (Figure 1).

Accordingly, delivering dCBT-I within a structured, reimbursed medical care context may provide a promising avenue to enhance accessibility and support the effective delivery. One such supportive care context that integrates medical supervision, structured daily routines, and physiological relaxation is crenotherapy. Crenotherapy is a medically supervised and reimbursed therapeutic intervention within the French healthcare system, involving structured thermal and balneotherapy treatments delivered in accredited medical centres. Beyond its historical use, crenotherapy offers a stable, supportive context that reinforces behavioural changes and promotes relaxation [33,34]. Beyond its historical use, recent work has highlighted the multiple roles of crenotherapy in contemporary healthcare, including stress regulation, pain management, and improvements in psychological well-being within a biopsychosocial framework [35]. Growing evidence suggests that crenotherapy may also be relevant for sleep-related outcomes. Improvements in sleep disturbances and insomnia-related complaints have been reported following such interventions [36,37], alongside beneficial effects on anxiety and stress-related symptoms [38,39], although most of the available evidence remains observational and warrants confirmation through controlled trials. Crenotherapy has been associated with reductions in anxiety and stress-related symptoms, which are conceptually and empirically linked to hyperarousal processes implicated in insomnia [40]. However, direct effects of crenotherapy on hyperarousal mechanisms have not been specifically established. In addition, the structured and immersive nature of crenotherapy stays, characterised by fixed daily routines and temporary separation from occupational and domestic stressors, may provide a favourable context for implementing behavioural recommendations central to CBT-I.

As a structured and reimbursed intervention prescribed in routine clinical practice, crenotherapy is broadly accessible across socioeconomic groups and has been associated with high levels of patient engagement and satisfaction [41]. However, although dCBT-I is an established treatment for chronic insomnia, treatment outcomes remain heterogeneous, and delivery context may influence adherence and hyperarousal-related processes. Crenotherapy provides a structured daily routine, medical supervision, and a relaxation-oriented environment, which may facilitate behavioural change and modulate anxiety-related mechanisms implicated in insomnia. Despite these potential benefits, evidence combining dCBT-I with crenotherapy remains scarce and largely indirect, with available studies primarily addressing related clinical objectives, such as benzodiazepine withdrawal in a crenotherapy context, rather than insomnia itself [42]. The primary aim of this multicentre proof-of-concept randomised controlled trial (RCT) was to evaluate whether embedding dCBT-I within a crenotherapy programme would yield greater reductions in insomnia severity compared with stand-alone dCBT-I. Secondary objectives were to assess differential effects on sleep parameters, pre-sleep arousal, anxiety and depressive symptoms, as well as engagement and satisfaction. Exploratory analyses further examined whether baseline characteristics were associated with treatment response among participants receiving dCBT-I within the crenotherapy care context. We hypothesised that delivery of dCBT-I within a structured and medically supervised crenotherapy context would yield greater reductions in ISI scores (an additional 2.5-point reduction) and anxiety-related measures, and potentially enhance adherence.

## 2. Method

### 2.1. Study Design

**Figure 2:**
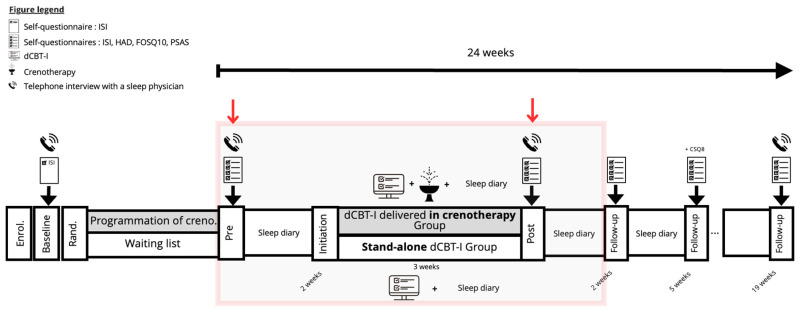
Study design: prospective, open-label, parallel-group randomised controlled trial comparing Digital Cognitive and Behavioural Therapy for Insomnia disorder (dCBT-I) delivered within a crenotherapy vs. dCBT-I delivered as a stand-alone intervention. Notes: Enrol.: Enrolment; Rand.: Randomization; Creno.: Crenotherapy; Pre: Pre-treatment; Post: Post-treatment; The red arrows indicate the ISI scores before and after treatment, which constitute the primary outcome.

This prospective RCT used a parallel-group design to compare dCBT-I delivered within a crenotherapy care context and stand-alone dCBT-I in participants with chronic insomnia disorder (Figure 2). Participants were aware of their group allocation (open-label design). Outcome measures were self-reported via the digital platform, and no blinded assessors were involved in post-treatment data collection. Eligibility interviews were conducted prior to randomisation. The study was conducted between 2019 and 2023 across five accredited crenotherapy centres specializing in sleep disorders in France (Saujon, Bagnères-de-Bigorre, Divonne-les-Bains, Néris-les-Bains and Ussat-les-Bains). Eligible individuals were identified through participant registries and recruited prospectively. All assessments were centralised and conducted by the coordinating centre at the Neuropsychopharmacological Research Platform of the Bordeaux University Hospital via standardised telephone interviews. The study was approved by the relevant ethics committee and registered under the French ID-RCB (n° 2019-A00043-54) and listed on ClinicalTrials.gov (NCT03991247).

### 2.2. Randomization

The random allocation sequence was generated prior to study initiation by the statistician of the Centre de Méthodologie et de Gestion des Données (Bordeaux PharmacoEpi CIC1401), independent from the clinical investigators. Participants were randomly assigned in a 1:1 ratio to either (i) dCBT-I delivered within a crenotherapy care setting or (ii) stand-alone dCBT-I delivered at home. Randomization was stratified by thermal centre to account for potential site-related variability. Allocation was implemented centrally through a secure web-based Interactive Web Response System (IWRS) integrated within the electronic case report form (eCRF), ensuring allocation concealment. Investigators responsible for participant screening and inclusion had no access to the randomization list. Participants were enrolled by the coordinating clinical team at SANPSY (sleep physicians and research coordinators), and assignment to intervention was performed automatically via the IWRS following confirmation of eligibility and informed consent. For each arm, the temporal sequence of procedures and the participant’s physical context at each stage of the study are summarised in Table 1, which presents a simplified study timeline.

### 2.3. Participants’ Inclusion

Participants were adults aged 18–80 who were registered for a crenotherapy stay scheduled within the next 12 months of enrolment, but no sooner than 16 weeks after inclusion. Eligibility was assessed during a standardised telephone interview with a board-certified sleep physician from the Neuro-Psychopharmacological Research Platform of the Bordeaux University Hospital. Inclusion criteria were: (1) age ≥ 18 years; (2) meeting DSM-5 criteria for insomnia disorder according to the sleep physician interview; (3) ISI score > 8 at baseline; and (4) access to a computer, tablet, or smartphone with internet connectivity. Exclusion criteria included: (1) presence or suspicion of another sleep disorder according to the sleep physician interview, based on the ICSD-3 diagnostic criteria; (2) initiation or recent dosage increase (within the past 2 months) of antidepressant medication; (3) initiation or recent dosage increase (within the past month) of anxiolytic, hypnotic, or neuroleptic treatment; (4) crenotherapy (within the past 6 months); (5) participation in another sleep-focused therapeutic program during the scheduled crenotherapy; (6) pregnancy or lactation; (7) legal guardianship (partial or full); (8) night, evening, or rotating shift work; and (9) transmeridian travel (±3 h) (within the past month). Participants allocated to the dCBT-I delivered within a crenotherapy care setting completed the digital intervention during their scheduled crenotherapy stay, whereas those allocated to the stand-alone dCBT-I completed the digital intervention prior to a later crenotherapy stay within the pre-specified 12-month period. Demographic and clinical characteristics, including age, height, weight, body mass index (BMI), sex, history of mental disorders (including major depressive disorder, generalized anxiety disorder, bipolar disorder, post-traumatic stress disorder, and burnout), previous use of crenotherapy, and current use of hypnotic medication, were collected during the same standardized telephone interview.

### 2.4. Procedures

Eligible participants were randomly assigned (1:1) to the dCBT-I delivered in crenotherapy Group or the stand-alone dCBT-I Group. Participants were then followed for 24 weeks, with assessments conducted via self-report questionnaires at six time points: baseline, pre-treatment, post-treatment, and at 2-, 5- and 19-week follow-up (Figure 2).

#### 2.4.1. Digital Cognitive and Behavioural Therapy for Insomnia Disorder

The dCBT-I was delivered via a secure web-based platform developed by the Information Technology Department of the Bordeaux University, which served both as an electronic sleep diary and as a tool for delivering personalised behavioural and cognitive recommendations. The platform was accessible via a secure web browser on computer, tablet, or smartphone. The interface used a responsive design that automatically adapted to different screen sizes, while maintaining identical therapeutic content and structure across devices. The intervention corresponded to an algorithm-guided digital CBT-I programme in which treatment recommendations were automatically generated based on sleep diary data, without real-time therapist involvement. This intervention model builds on our laboratory’s prior experience with dCBT tools for insomnia disorder [43,44], and is conceptually aligned with prior short-term internet-delivered CBT-based self-help programs combining sleep restriction and stimulus control (PROPERSOM protocol), following established recommendations for the development of sleep diaries [8] and the international recommendation for CBT-I [45,46].

Participants logged in daily with individual credentials to complete the sleep diary across a 10-week period, covering a 2-week pre-treatment phase, a 3-week intervention phase, and 2-week and 5-week post-treatment follow-ups. During the 3-week intervention phase, participants received structured digital content organised into sequential therapeutic modules delivered weekly, including (i) sleep hygiene education, (ii) stimulus control procedures to restore bed–sleep associations, and (iii) sleep restriction therapy with automated weekly adjustments. The 3-week intervention phase was deliberately aligned with the standard duration of crenotherapy care within the French healthcare system, allowing synchronisation between the digital intervention and the medical care context for participants allocated to the crenotherapy group. Sleep windows were initially defined based on baseline sleep diary data and subsequently adjusted weekly according to sleep efficiency calculated over a 7-day rolling average, following standard CBT-I sleep restriction principles. Specifically, the platform updated the recommended time in bed each week by either adding 15 min, reducing 15 min, or maintaining the previous bedtime recommendation, based on the 7-day mean sleep efficiency. When insufficient diary data were available to compute sleep efficiency, the previous sleep schedule was maintained until adequate diary completion allowed recalculation.

No synchronous therapist guidance was provided during the intervention. Operational clinical oversight was provided through the coordinating study team (sleep physician and research staff), who could be contacted by participants throughout the study in case of technical or medical difficulties. In addition, for participants allocated to the crenotherapy arm, weekly onsite medical consultations during the 3-week stay supported treatment tolerance and reinforced understanding of the behavioural recommendations.

#### 2.4.2. Crenotherapy

All accredited crenotherapy medical centres use mineral waters officially recognised by the French National Academy of Medicine and the French Ministry of Health, with geological validation by the Bureau of Geological and Mining Research (BRGM). All centres follow standardised reimbursed therapeutic protocols defined by a national agreement with the French National Health Insurance (CNAM), ensuring uniform treatment delivery across sites. The crenotherapy intervention was delivered over a standard 3-week period, corresponding to the duration of reimbursed crenotherapy stays within the French healthcare system, and consisted of two components. First, participants received weekly medical consultations throughout the intervention (three 15 min consultations in total), during which the physician ensured that the treatment was well tolerated, adjusted the thermal care as needed to the patient’s condition, and promoted a state of relaxation during the crenotherapy interventions. Second, participants completed the standardised “PSY4” treatment protocol, which comprised three daily hydrotherapy sessions in the morning over an 18-day period: bubbling baths (10 min), thermal showers (3 min), and thermal pool sessions (10 min). Water temperatures were maintained at 31–33 °C for pool sessions and 35–38 °C for all other hydrotherapy procedures. In addition, a 20-min underwater massage was administered once every two days, by a licensed physiotherapist.

### 2.5. Outcomes

#### 2.5.1. Insomnia Symptoms

Severity of insomnia symptoms and related daytime functional impact were assessed using the Insomnia Severity Index (ISI; [47], validated in French [48]). The ISI is a validated 7-item self-report questionnaire rated from 0 to 4, yielding a total score ranging from 0 to 28. Severity categories are defined as follows: 0–7 (no insomnia), 8–14 (mild), 15–21 (moderate), and 22–28 (severe).

#### 2.5.2. Subjective Sleep Parameters

Electronic sleep diary data enabled daily monitoring of key sleep behaviour parameters [8]: Time-In-Bed (TIB, the time elapsed between bedtime and rise time), Wake After Sleep Onset (WASO, the total time spent awake between bedtime and rise time), Total Sleep Time (TST, the time elapsed between sleep time and wake time, minus WASO, or, the total time spent asleep between sleep time and wake time), Sleep Efficiency (SE, the percentage of TST relative to TIB), Sleep Onset Latency (SOL, the time elapsed between bedtime and sleep time), Late Insomnia (LI, time elapsed between final awakening and rise time).

#### 2.5.3. Sleep Functioning

The impact of sleep disturbances on daily functioning was assessed using the Functional Outcomes of Sleep Questionnaire-10 (FOSQ-10; [49]). The FOSQ is a 10-item self-report questionnaire rated from 0 to 4, yielding a total score ranging from 0 to 40, with higher scores indicating greater impairment in daily functioning. A threshold of 11.5, based on the sample’s median, was used to distinguish between low and high impairment.

#### 2.5.4. Pre-Sleep Arousal

Pre-sleep arousal, encompassing cognitive and somatic hyperactivation, was assessed using the Pre-Sleep Arousal Scale (PSAS; [50]). The PSAS is a 16-item self-report questionnaire rated from 1 to 5, yielding a total score ranging from 16 to 80. A threshold of 26 for the cognitive subscale, 15 for the somatic subscale, based on the sample’s median, was used to distinguish between low and high pre-sleep arousal.

#### 2.5.5. Anxiety and Depressive Symptoms

Anxiety and depressive symptoms were assessed using the Hospital Anxiety and Depression Scale (HADS; [51]). The HADS is a 14-item self-report questionnaire rated from 0 to 3, with two subscales, anxiety (HADS-A) and depression (HADS-D), yielding a total score ranging from 0 to 21 for each subscale. Scores ≤ 7 indicate no symptoms, 8–10 reflect mild symptoms, and ≥11 reflect clinically significant symptoms.

#### 2.5.6. Engagement and Satisfaction Evaluation

Intervention engagement was measured by the average number of days the sleep diary was completed, during 10 weeks, starting at pre-treatment. Participant satisfaction was measured using the Client Satisfaction Questionnaire (CSQ-8; [52]). The CSQ-8 is an 8-item self-report questionnaire rated from 1 to 4 yielding a total score ranging from 8 to 32, with higher scores indicating greater satisfaction. A threshold of 26.5, based on the sample’s median, was used to distinguish between lower and higher levels of satisfaction.

### 2.6. Sample Size

Sample size was calculated based on the primary outcome: change in ISI scores from pre- to post-treatment between stand-alone dCBT-I and dCBT-I delivered within a crenotherapy care setting. Assuming a two-sided alpha of 0.05 and 80% power, the calculation was based on an expected between-group difference of 2.5 points on the ISI and a standard deviation of 4.8. This expected effect size was informed by prior work examining reductions in insomnia severity in multimodal care contexts, including crenotherapy-associated reductions in hyperarousal and anxiety [40] and consistent with values reported in previous CBT-I trials and digital interventions [17]. Based on these assumptions, a total sample size of 100 participants (50 per group) was required. To account for an anticipated dropout rate of approximately 20%, which is consistent with attrition rates commonly reported in randomised controlled trials of digital psychological interventions [23,53], the final target sample size was set at 120 participants (60 per group).

### 2.7. Statistical Analysis

Analyses were conducted on randomised participants who completed the post-treatment assessment and had available pre- and post-treatment data (complete-case analysis). No formal intention-to-treat (ITT) analysis including all randomised participants (*n* = 120) was performed. Demographic and baseline clinical characteristics (including age, sex, height, weight, BMI, history of mental disorders, previous crenotherapy, and use of hypnotic medication) were collected at the time of enrolment and were summarised by group (Table 2). Between-group comparisons at baseline were performed using Student’s *t*-test for quantitative variables and Chi-square tests for categorical variables.

The primary endpoint was defined a priori as the between-group difference in change in ISI score from pre- to post-treatment at the specified post-treatment time point. For the primary objective, within-group changes in ISI scores (pre- to post-treatment) were computed using paired *t*-tests. Between-group differences in ISI change scores were assessed using independent *t*-tests to evaluate the potential additive effect of crenotherapy delivered within dCBT-I. This primary analysis was conducted on participants with available pre- and post-treatment ISI data. Participants with missing post-treatment outcome data were not included in change score analyses.

For the first secondary objective, to examine changes in sleep-related and mental symptoms following dCBT-I delivered within a crenotherapy care setting and stand-alone dCBT-I, within- and between-group analyses (paired and independent *t*-tests) were computed for additional outcomes, including sleep behaviours, sleep functioning, pre-sleep arousal, and anxiety and depressive symptoms. Day-to-day variations in sleep behaviours were explored through graphical visualisation.

For the second secondary objective, to assess whether a comprehensive set of baseline characteristics predict treatment outcomes in dCBT-I delivered within crenotherapy, exploratory stratified analyses were used to identify predictors of treatment outcome. Potential predictors were categorised using median splits, with the exception of sex, which was treated as a binary variable. Categorisation thresholds were as follows: age (>60 years), insomnia severity (ISI > 15), pre-sleep arousal (PSAS cognitive > 26; somatic > 15), sleep functioning (FOSQ-10 > 11.5), and anxiety and depressive symptoms (HADS-A or -D > 11). Treatment outcome (defined by the difference between pre-post ISI scores) was described within each stratum (e.g., treatment effect of dCBT-i delivered within crenotherapy among female participants). Interactions between potential predictors and the treatment outcome were computed using independent *t*-tests (e.g., sex-based differences in the treatment effect of dCBT-I delivered within crenotherapy were examined by comparing outcomes between female and male participants). No correction for multiple comparisons was applied to exploratory subgroup analyses.

For the third secondary objective, to assess sleep diary engagement and participant satisfaction, between groups using independent *t*-tests were computed.

All statistical analyses were conducted using JAMOVI (Version 2.6.25.0) and GraphPad Prism (Version 10.5.0). Normality was assessed for all continuous variables, and significance was set at a two-sided alpha level of 0.05.

Participants were considered dropouts if post-treatment outcome assessments, including the primary ISI measure, were not completed. Missing sleep diary entries during the intervention alone did not constitute dropout status. To assess the robustness of the primary outcome in the presence of missing post-treatment data, a sensitivity analysis using a baseline observation carried forward (BOCF) approach was performed. In this conservative scenario, missing post-treatment ISI scores were replaced by baseline values, assuming no change in insomnia severity among participants with missing outcome data.

## 3. Results

### 3.1. Participants

**Figure 3:**
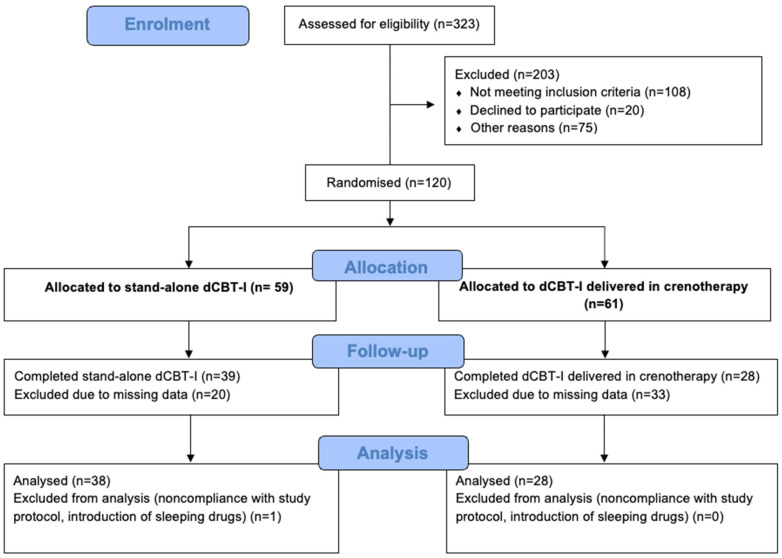
Participants flow through the trial.

Figure 3 presents the participant flow through the trial, including enrolment, randomization, intervention allocation, follow-up, and analysis. At baseline, the two groups were comparable with no statistically significant differences in demographic or clinical characteristics (Table 2). Most participants were women, and a high proportion had a history of mental disorders, prior exposure to crenotherapy, and regular use of hypnotic medication. Overall, 23.9% of participants met the criteria for severe insomnia disorder (ISI > 21). Of the 120 participants initially randomised, 66 had complete pre- and post-treatment outcome data and were included in the primary change-score analysis. Comparisons of baseline sociodemographic, clinical, and insomnia-related characteristics revealed no significant differences between completers and participants with dropout or missing outcome data (all *p* > 0.05), suggesting a low likelihood of systematic attrition bias (Supplementary Materials (Table S1)).

### 3.2. Impact of Crenotherapy on Insomnia Symptoms

**Figure 4:**
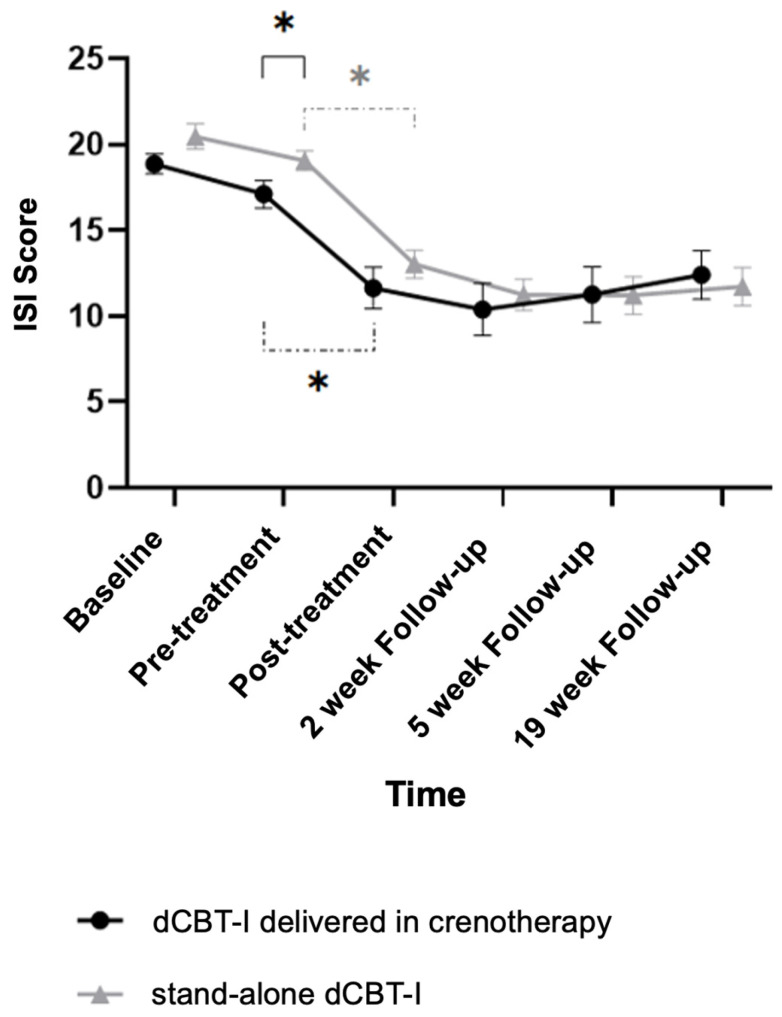
Changes in insomnia symptoms from baseline to the 19-week post-treatment follow-up in both groups: dCBT-I delivered within crenotherapy and stand-alone dCBT-I. The solid bracket indicates significant between-group difference, while the two dash brackets denotes a significant within-group difference. Asterisks indicate statistically significant differences (*p* < 0.05).

Figure 4 illustrates changes in insomnia symptom severity from baseline to the 19-week follow-up in both groups. As shown in Table 3, no significant between-group difference was observed for the primary outcome (pre-to-post ISI change), nor at follow-up. Although pre-treatment ISI scores were significantly lower in the dCBT-I delivered within crenotherapy group compared with the stand-alone group (17.11 ± 4.24 vs. 19.08 ± 3.49), post-treatment and follow-up scores did not differ significantly between groups. Results from the BOCF sensitivity analysis were consistent with the primary complete-case analysis and did not reveal any significant between-group difference in the change in ISI scores. Mean ISI decreased from 18.64 to 14.79 in the stand-alone dCBT-I group and from 18.07 to 15.64 in the crenotherapy group. The between-group comparison remained non-significant (t = −0.80, *p* = 0.425), confirming the robustness of the main findings. Within-group analyses showed significant reductions in insomnia symptoms from pre- to post-treatment in both groups (dCBT-I delivered within crenotherapy: 17.11 ± 4.24 to 11.64 ± 6.40; stand-alone dCBT-I: 19.08 ± 3.49 to 13.03 ± 4.94). At post-treatment, 55.26% of participants in the stand-alone group and 46.43% in the crenotherapy group achieved a clinically meaningful response (≥8-point reduction in ISI), with no significant between-group difference (*p* = 0.356). Remission rates (ISI < 8) were 13.16% and 32.14%, respectively, although this difference did not reach statistical significance (*p* = 0.062).

### 3.3. Impact of Crenotherapy on Other Sleep Parameters and Mental Symptoms

**Figure 5:**
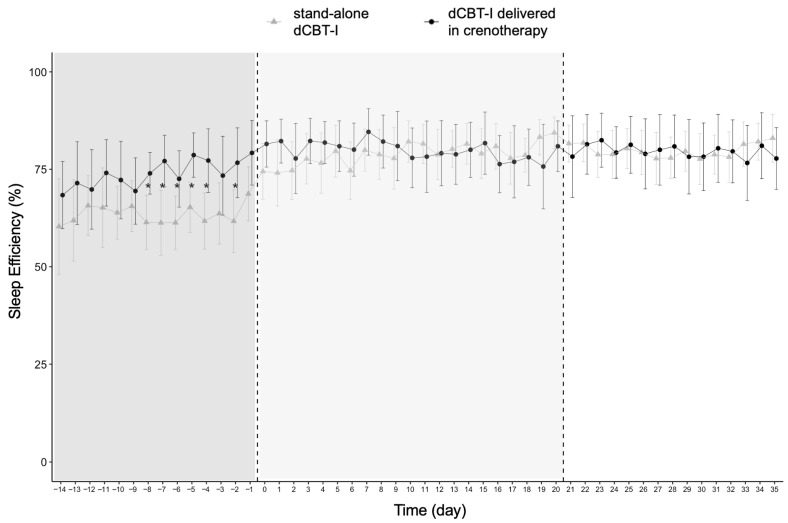
Day-to-day evolution of Sleep Efficiency across three phases (pre-treatment in dark grey, during treatment in light grey, and post-treatment in white) for both the stand-alone dCBT-I group (grey line) and the dCBT-I delivered within crenotherapy group (black line). Asterisks indicate time points at which between-group differences reached statistical significance (*p* < 0.05). Pre-intervention differences are presented descriptively. Additional sleep behaviour parameters, modelled using the same approach, are presented in the Supplementary Materials.

Table 3 reports within- and between-group effects on sleep-related behaviours, sleep functioning, pre-sleep arousal, and anxiety and depressive symptoms. Importantly, between-group comparisons revealed a significantly greater reduction in anxiety symptoms in the dCBT-I delivered within crenotherapy group compared with stand-alone dCBT-I. No other significant between-group differences over time were observed. Within-group analyses showed significant improvements from pre- to post-treatment in both groups in SE, SOL, LI, TIB, sleep functioning, as well as anxiety and depressive symptoms, while TST remained unchanged. WASO and pre-sleep arousal significantly decreased only in the stand-alone dCBT-I group. Figure 5 illustrates the day-to-day evolution of SE (%) in both groups, showing an early improvement in the dCBT-I delivered within crenotherapy group during the days preceding the start of crenotherapy. Additional day-to-day sleep behaviour trajectories are provided in Supplementary Materials.

### 3.4. Impact of Baseline Characteristics on Insomnia Symptoms

**Figure 6:**
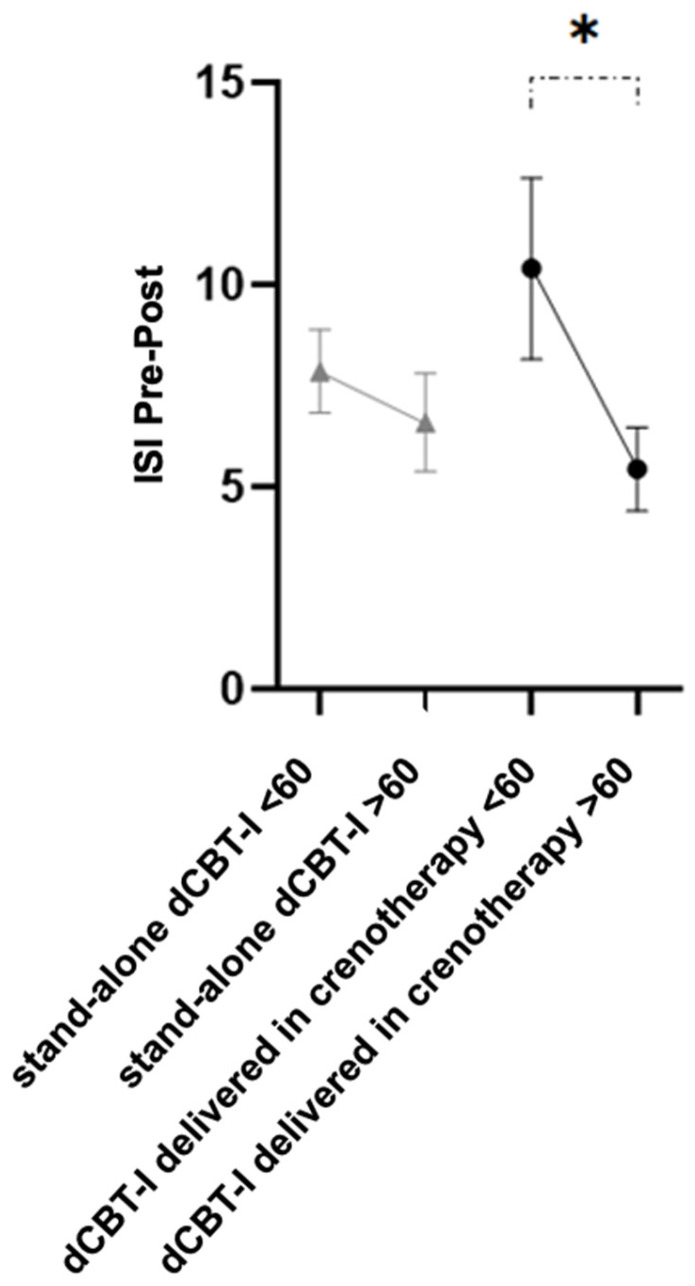
Pre-post Insomnia Severity Index scores differences across age and intervention group (higher values indicating greater reductions in the severity of insomnia symptoms). Asterisks indicate statistically significant differences (*p* < 0.05).

As pre-specified in the Methods, age was categorised using a median split (>60 years) for exploratory stratified analyses. These subgroup analyses were not powered for formal interaction testing and should therefore be interpreted as hypothesis-generating. These analyses suggested that age was associated with differential changes in insomnia symptom severity according to crenotherapy delivery context. Indeed, among participants receiving dCBT-I delivered within crenotherapy, those under 60 showed a significantly greater reduction (10.40 ± 7.07) than their older counterparts (5.44 ± 4.38; *p* = 0.030; Figure 6). No significant associations were found for other potential predictors of treatment effect (sex, baseline insomnia symptom severity, baseline pre-sleep arousal, baseline sleep functioning, or baseline anxiety and depressive symptoms).

### 3.5. Satisfaction

Regarding sleep diary completion, the mean number of days filled out over the 10-week period (70 days) was 67.70 ± 2.50 in the stand-alone dCBT-I group and 65.59 ± 9.74 in the dCBT-I delivered within crenotherapy group, with no significant between-group difference (*p* = 0.211). Among all randomised participants (*n* = 120), 90% initiated use of the digital platform by completing at least one sleep diary entry. During the 3-week intervention phase, 80.83% logged into the platform and recorded at least one diary entry. Initiation rates were calculated across the full randomised sample, as group-level disaggregated data were not available from the extracted dataset. Mean satisfaction scores were 24.84 ± 5.29 in the dCBT-I delivered within a crenotherapy care setting group and 26.85 ± 4.41 in the stand-alone dCBT-I group, with no significant difference observed (*p* = 0.148).

## 4. Discussion

To our knowledge, this is among the first RCT to evaluate the effectiveness of dCBT-I on insomnia symptom severity when delivered within a crenotherapy care setting. Both groups, those receiving dCBT-I delivered within crenotherapy and those receiving stand-alone dCBT-I, showed significant improvements in severity of insomnia symptoms as well as across multiple sleep-related parameters, including Sleep Efficiency (SE), Sleep Onset Latency (SOL), Late Insomnia (LI) and total Time spent In Bed (TIB). These findings are consistent with the established effectiveness of dCBT-I for chronic insomnia disorder reported in previous randomised controlled trials and meta-analyses [17,54,55]. In the present study, both groups receiving dCBT-I showed significant within-group improvements, although no comparison was made against a non-active control condition. However, no additional benefit of crenotherapy was observed on insomnia symptom severity at the group level. One possible explanation for the absence of an additional effect of crenotherapy on ISI scores is the substantial improvement observed in both groups following dCBT-I. The reduction in ISI severity from the moderate range (~19 points) to the mild range (~11–13 points) represents a clinically meaningful change. It is therefore possible that the strong therapeutic impact of dCBT-I limited the capacity to detect an incremental benefit of crenotherapy on insomnia severity, suggesting a potential ceiling effect.

A possible anticipatory effect linked to crenotherapy should also be considered. A descriptive increase in sleep efficiency was observed prior to the start of the crenotherapy stay. In addition, pre-treatment ISI scores were significantly lower in the dCBT-I delivered within crenotherapy group compared with the stand-alone group. Although these pre-intervention patterns were not formally tested using longitudinal slope analyses, they may reflect expectation-related mechanisms associated with the upcoming thermal stay. As slope-based comparisons were not pre-specified in the statistical analysis plan, these observations should be interpreted descriptively and as hypothesis-generating. Future studies using mixed-effects modelling in larger, adequately powered samples would allow formal testing of such expectation-related trajectories. This suggests that psychological factors such as expectation and preparatory engagement may have played a role in early symptom reduction [56,57].

Importantly, exploratory secondary analyses indicated that when implemented within a crenotherapy care setting, dCBT-I was associated with greater reductions in anxiety symptoms compared with stand-alone dCBT-I. Although anxiety symptoms showed greater reductions in the crenotherapy group, changes in pre-sleep arousal (PSAS) did not differ significantly between groups. This suggests that the potential added value of crenotherapy may be more closely related to broader anxiety regulation than to specific reductions in cognitive or somatic pre-sleep arousal as measured in this study. This finding aligns with results from the “Stop TAG” RCT [58], which highlighted the anxiolytic potential of crenotherapy. Beyond its role as a supportive care context, crenotherapy may exert specific therapeutic effects, particularly in relation to anxiety regulation [33,36] and consistent with our prior preliminary findings [40]. This finding is particularly noteworthy given the central role of anxiety and hyperarousal in insomnia pathophysiology, and aligns with prior evidence suggesting anxiolytic effects of crenotherapy. Such effects are particularly relevant for insomnia phenotypes characterised by elevated cognitive and physiological hyperarousal, where anxiety plays a central role in sleep symptom maintenance [59]. Moreover, the ISI primarily captures perceived insomnia severity and daytime impairment, but may be less sensitive to contextual or physiological modulation occurring through environmental or relaxation-based mechanisms. This may partly explain why crenotherapy showed differential effects on anxiety symptoms without translating into additional ISI improvement.

Furthermore, exploratory secondary analyses suggested that among participants under the age of 60, those receiving dCBT-I delivered within crenotherapy showed a significantly greater reduction in insomnia symptoms than their counterparts receiving stand-alone dCBT-I, a pattern not observed in older participants. However, these exploratory subgroup findings were not the primary endpoint and the study was not powered to detect interaction effects. They should therefore be interpreted cautiously and considered as hypothesis-generating signals requiring confirmation in future pre-registered and adequately powered trials. Although these findings should be interpreted cautiously, they suggest that crenotherapy may provide added contextual benefits, potentially through temporary removal from everyday stressors related to home and occupational environments [33], particularly among individuals with specific baseline characteristics, such as anxiety symptoms, which are commonly comorbid with insomnia disorder [60]. One possible explanation for the observed age-related effect is that younger participants may have benefited more from being temporarily extracted from work-related pressures, especially in cases of professional burnout [61]. However, no occupational or socioeconomic data were collected to explore this hypothesis further. Alternatively, insomnia in older adults may more frequently reflect age-related or maintenance-type sleep disturbances, potentially driven by biological or medical factors that are less responsive to contextual or environmental changes [62]. In addition, the structured temporal framework of the crenotherapy setting (e.g., fixed wake-up and treatment times) may support the adoption of behavioural recommendations central to CBT-I [63]. These observations are consistent with findings from circadian intervention studies, which emphasise the role of routine in promoting sleep–wake regulation [64,65].

Together, these findings underscore the potential of embedded care models (Figure 1) to enhance treatment outcomes and guide the development of more personalised therapeutic approaches, tailored to the phenotypic profiles of individuals with insomnia disorder [66]. A distinctive contribution of this study lies in its evaluation of an embedded delivery model of dCBT-I, distinct from traditional stand-alone or fully automated digital interventions [67]. In this embedded model, dCBT-I delivered within a structured reimbursed medical care context, such as crenotherapy, is situated between fully automated digital formats and conventional face-to-face interventions. Within this framework, therapeutic support extends beyond digital guidance to encompass immersive, routine-based, and person-centred care. This perspective aligns with broader developments in digital psychiatric rehabilitation, where integrated care models combine technological tools with structured therapeutic environments to enhance contextualisation and patient engagement across mental health conditions [68].

While several contextual features of this model may be transferable beyond France, the generalisability of the present findings should nevertheless be interpreted in light of the specific organisation of crenotherapy within the French healthcare system. Crenotherapy is medically prescribed, standardised, and publicly reimbursed, conditions that may not be directly replicable in other settings. Nevertheless, several contextual features of the embedded model may be transferable beyond France, including body care, structured daily routines, supervised medical oversight, temporary disengagement from occupational and domestic stressors, and delivery within a residential therapeutic environment. Future research should clarify whether these contextual elements, rather than crenotherapy as a modality per se, account for the subgroup effects observed. By leveraging coordinated medical infrastructure and direct community access through healthcare providers, this embedded model may help support the delivery and uptake of dCBT-I within routine care pathways. Such contextual factors are particularly relevant for digital interventions, where engagement and implementation can be challenging and heterogeneous [69,70], and where treatment outcomes have been shown to vary substantially according to delivery modalities and care contexts rather than digital format alone [24,25]. Although the spa environment is primarily associated with relaxation, engagement indicators did not suggest increased treatment burden in the crenotherapy group. However, the coexistence of behavioural sleep restriction within a relaxation-oriented setting may warrant further qualitative investigation in future studies.

Future research should also continue to explore embedded dCBT-I delivery models within stepped and integrated care frameworks, in alignment with recent recommendations supporting scalable, guideline-based digital interventions for insomnia [21]. In parallel, these efforts should be coupled with public health strategies aimed at expanding access to evidence-based insomnia treatments, particularly through digitally supported solutions tailored to real-world care contexts [71].

## 5. Limitations

This study presents several limitations that should be acknowledged when interpreting the results. First, there was a high rate of dropout or missing outcome data (45%), partly attributable to the extended inclusion period and external disruptions, notably those related to the COVID-19 pandemic. Notably, this dropout rate was comparable to those reported in other randomised controlled trials of digital and internet-delivered psychological interventions (e.g., [53]. Most importantly, participants with dropout or missing outcome data were comparable to others in terms of sociodemographic, clinical, and insomnia-related characteristics (Supplementary Materials (Table S1)), which reduces the likelihood of attrition bias. Furthermore, the relatively small final sample size, combined with the substantial attrition observed during follow-up, may have reduced the statistical power to detect modest between-group effects. In addition, the high dropout rate may have weakened the internal validity of the trial and partially weakened the balance achieved through initial randomization. A conservative sensitivity analysis using BOCF yielded results consistent with the primary analysis, although this approach assumes no improvement among participants with missing data and may underestimate treatment effects. Potential barriers may have included the cognitive and behavioural demands of sustained sleep diary completion, the coexistence of sleep restriction with daytime thermal procedures, and contextual factors related to the extended recruitment period. Future trials should integrate real-time adherence monitoring and adaptive engagement strategies.

In addition, the final analysed sample (*n* = 66) did not reach the initially planned target of 100 participants required by the a priori power calculation, resulting in reduced statistical power to detect the expected between-group effect size. In addition, detailed module-level adherence metrics and early dropout trajectories were not prospectively defined and could not be fully reconstructed from the available database, limiting the granularity of engagement analyses. Moreover, the primary analyses were conducted on participants with complete outcome data rather than using a full ITT framework including all randomised participants, which may have introduced bias and further reduced statistical power. It is possible that participants who completed the protocol were more motivated or treatment-responsive, potentially leading to an overestimation of treatment effects. However, the extent and structure of missing data, combined with the relatively small final sample size, limited the feasibility and statistical stability of such ITT modelling, particularly with respect to random-effects estimation. Overall, these findings underscore the importance of implementing targeted retention strategies, particularly in real-world care settings, where attrition remains a well-documented challenge. Future studies could benefit from reinforcing follow-up procedures and tailoring engagement efforts to specific participant profiles to improve adherence and data completeness.

Second, the potential anticipatory effect linked to crenotherapy could not be adequately assessed. Since 78.9% of participants in the crenotherapy group had already experienced a similar stay, early improvements may have occurred prior to the intervention itself. However, no data were collected to further explore this effect. Future research should integrate qualitative and quantitative assessments [72,73,74] to investigate these anticipatory processes and their impact on outcomes, particularly within embedded approaches to insomnia care [75]. In addition, because dCBT-I was delivered at different time points relative to the crenotherapy stay across groups, potential seasonal effects (e.g., variations in daylight exposure, occupational stress, or mood fluctuations) cannot be entirely excluded and may have influenced treatment responses.

Third, the absence of complementary data, including objective sleep measures (e.g., actigraphy), physiological indicators (e.g., electrodermal activity) and sociodemographic variables, limits the depth of interpretation. Including these dimensions in future research would help clarify underlying mechanisms and better characterise patient subgroups likely to benefit from combined interventions. In addition, although analyses relied on pre-to-post comparisons using *t*-tests in line with the pre-specified outcome, alternative approaches such as Linear Mixed Models may have provided a more flexible framework to account for repeated measures and missing data. Nevertheless, the relatively small sample size may have limited the stability of random-effects estimation, and larger samples would be required to fully leverage such models.

Finally, the study design does not allow the specific contribution of crenotherapy itself to be disentangled from that of dCBT-I. To more precisely assess the unique and interactive effects of dCBT-I when delivered within a crenotherapy context, future studies should adopt a factorial design including three groups: dCBT-I alone, crenotherapy alone, and dCBT-I delivered within crenotherapy. Such a design would allow a clearer evaluation of additive or synergistic effects and strengthen conclusions regarding the specificity of the embedded intervention model. A future factorial trial (dCBT-I alone vs. crenotherapy alone vs. combined intervention) should predefine the primary outcome as the between-group difference in change in ISI at the end of the intervention phase (e.g., 3 weeks), with sample size estimation based on a clinically meaningful incremental effect. Given the substantial improvements typically observed with dCBT-I and the resulting potential plateau effect on ISI, the added benefit attributable to contextual embedding is likely to be modest at the group level. Accordingly, effect size assumptions could be based on a conservative clinically meaningful difference in the range of ~2.5–3 ISI points, consistent with published estimates of minimal clinically important change for the ISI in clinical populations (e.g., MCID ≈ 2.4–2.6; [76], while acknowledging that larger within-person improvements have also been proposed in primary insomnia samples [77,78]. Retention strategies should be prospectively planned, including reminder systems, structured follow-up contacts, and predefined engagement monitoring, while also taking into account the role of behavioural regulation and contextual anchoring in sustaining therapeutic change. In addition, potential moderators such as age, baseline anxiety, pre-sleep arousal, and indicators of daily routine stability or behavioural organisation should be pre-registered and tested using interaction terms in adequately powered models.

## 6. Conclusions

This RCT did not demonstrate a superior effect of delivering dCBT-I within a crenotherapy setting on overall insomnia severity compared with stand-alone dCBT-I. However, this study supports the effectiveness of dCBT-I for chronic insomnia and provides new insights into its integration within a structured reimbursed medical care context. While no overall group-level effect of crenotherapy on insomnia severity was observed, exploratory findings suggest that delivery of dCBT-I within a crenotherapy care setting may be associated with differential benefits for specific subgroups, including adults under retirement age and individuals presenting with anxiety symptoms. These exploratory subgroup findings should not be interpreted as sufficient evidence to modify current clinical practice but rather as preliminary signals to inform future confirmatory trials.

Taken together, these findings highlight the potential of embedded care models, where digital therapies are delivered within supportive clinical environments, not necessarily to enhance effectiveness per se, but to support implementation and contextual fit within personalised care pathways. The added value of contextual embedding may not lie in universally amplifying insomnia severity outcomes, but rather in optimising implementation, enhancing engagement, and facilitating benefits in clinically relevant subgroups.

From a practical perspective, clinicians working in spa or rehabilitation settings may consider integrating dCBT-I within structured care programmes. Conversely, when initiating dCBT-I, embedding the intervention within a supportive therapeutic framework may represent a pragmatic strategy for patients with anxiety-related or stress-sensitive insomnia phenotypes.

Future research should further refine phenotypic profiling and clarify anticipatory and contextual mechanisms to inform more targeted, scalable and accessible insomnia care strategies in real-world settings.

## Figures and Tables

**Table 1:** Temporal sequence of enrolment, baseline assessment, intervention, and follow-up procedures in the stand-alone dCBT-I group and the dCBT-I delivered within crenotherapy group, specifying participant physical context (home vs. thermal centre) at each phase.

Phase	Stand-Alone dCBT-I	dCBT-I Delivered in Crenotherapy	Participant Location
Screening (E1–E2)	Eligibility assessment and consent	Eligibility assessment and consent	Home
Baseline (E2)	ISI	ISI	Home
Pre-treatment period (W0–W2)	ISI, secondary outcomes and 2 weeks sleep diary	ISI, secondary outcomes and 2 weeks sleep diary	Home
Intervention (W2–W5)	3-week dCBT-I program and sleep diary	3-week dCBT-I program and sleep diary	Home (stand-alone)/Thermal centre (in crenotherapy group)
Primary endpoint (W5: Post-treatment)	ISI and secondary outcomes	ISI and secondary outcomes	Home
Follow-up (W7, W10, W24, M6)	ISI and secondary outcomes	ISI and secondary outcomes	Home

Notes: E1–E2: single telephone eligibility assessment prior to randomization; E2: 62.3 ± 82.0 days (median = 55 days; range = 0–501 days) before W0; W0: Week 0; W2: Week 2; W5: Week 05; W7: Week 7; W10: Week 10; W24: Week 24; M6: Month 6.

**Table 2:** Sociodemographic, clinical and insomnia disorder characteristics at baseline in the stand-alone dCBT-I group and the dCBT-I delivered within crenotherapy group.

	Stand-Alone dCBT-I Group (*n* = 38)	dCBT-I Delivered in Crenotherapy Group (*n* = 28)
Age, *mean* ± SD	58.13 ± 7.11	60.61 ± 10.93
Height, *mean* ± SD	163.39 ± 7.83	166.75 ± 9.28
Weight, *mean* ± SD	67.66 ± 15.46	65.07 ± 14.32
BMI, *mean* ± SD	25.28 ± 5.22	23.27 ± 4.01
Female, *n*, (%)	34 (89.47%)	23 (82.14%)
History of mental disorders, *n*, (%)	25 (65.79%)	16 (57.14%)
Previous crenotherapy, *n*, (%)	30 (78.95%)	21 (75%)
Hypnotic medication, *n*, (%)	21 (55.26%)	15 (53.57%)
ISI score, *mean* ± SD	20.45 ± 3.56	18.86 ± 3.91
Severe insomnia disorder (ISI > 21), *n*, (%)	14 (36.84%)	8 (28.57%)

Notes: BMI: Body Mass Index; ISI: Insomnia Severity Index; ISI > 21 indicates severe insomnia disorder.

**Table 3:** Within-group and between-group differences in sleep parameters and anxiety and depressive symptoms across stand-alone dCBT-I and dCBT-I delivered within crenotherapy.

	Within Group								Between Groups			
	Stand-Alone dCBT-I				dCBT-I Delivered in Crenotherapy				Stand-Alone dCBT-I vs. dCBT-I Delivered in Crenotherapy			
									Pre		Post	
	Pre	Post	Cohen’s d	*p* Value	Pre	Post	Cohen’s d	*p* Value	Delta	*p* Value	Delta	*p* Value
ISI	19.08 ± 3.49	13.03 ± 4.94	1.25	**<0.001**	17.11 ± 4.24	11.64 ± 6.4	0.93	**<0.001**	1.97	**0.044**	1.39	0.328
SE	64.17 ± 14.26	79.40 ± 12.88	−1.57	**<0.001**	74.28 ± 15.48	80.21 ± 16.96	−0.49	**0.025**	−10.11	0.064	−0.81	0.831
SOL	54.16 ± 47.29	26.04 ± 17.39	0.71	**<0.001**	36.19 ± 22.12	26.80 ± 16.42	0.43	**0.044**	17.91	0.076	−0.76	0.865
TST	393.06 ± 78.81	394.69 ± 77.60	−0.03	0.846	408.78 ± 86.76	416.94 ± 95.11	−0.19	0.364	−15.72	0.502	−22.25	0.321
LI	54.27 ± 36.71	26.92 ± 28.15	0.95	**<0.001**	36.42 ± 32.07	22.85 ± 21.19	0.54	**0.015**	17.85	0.056	4.07	0.547
TIB	501.14 ± 61.51	447.47 ± 71.92	1.10	**<0.001**	481.90 ± 71.04	466.59 ± 79.41	0.48	**0.028**	19.24	0.239	−19.12	0.334
WASO	68.36 ± 42.11	34.12 ± 27.91	1.00	**<0.001**	47.74 ± 38.12	35.71 ± 33.17	0.29	0.170	20.62	0.092	−1.59	0.841
FOSQ10	11.81 ± 2.61	12.78 ± 3.14	−0.38	**0.028**	12.18 ± 4.24	13.50 ± 4.36	−0.51	**0.015**	−0.37	0.651	−0.72	0.251
PSAS-C	26.76 ± 6.95	22.62 ± 8.23	0.62	**<0.001**	22.91 ± 8.40	20.22 ± 8.12	0.40	0.067	3.85	0.106	2.40	0.216
PSAS-S	15.34 ± 5.54	13.94 ± 4.95	0.36	**0.041**	16.00 ± 5.34	15.65 ± 5.95	0.06	0.768	−0.66	0.516	−1.71	0.212
HADS-A	12.71 ± 4.23	10.71 ± 4.69	0.70	**<0.001**	11.23 ± 3.23	8.27 ± 2.89	0.87	**<0.001**	1.48	0.165	2.44	**0.030**
HADS-D	8.65 ± 3.70	7.24 ± 4.10	0.48	**0.006**	7.59 ± 5.04	5.37 ± 4.32	0.56	**0.008**	1.06	0.304	1.87	0.058

Notes: ISI: Insomnia Severity Index; SE: Sleep Efficiency; SOL: Sleep Onset Latency; TST: Total Sleep Time; LI: Late Insomnia; TIB: Total time spent In Bed; WASO: Wake After Sleep Onset; FOSQ10: Functional Outcome Sleep Questionnaire; PSAS-C/S: Pre-Sleep Arousal Scale—subscale Cognitive/Somatic; HADS-A/D: Hospital Anxiety and Depression Scale—subscale Anxiety/Depression. Values in bold indicate statistically significant differences (*p* < 0.05).

## Data Availability

The data that support the findings of this study are available on request from the corresponding author. The data are not publicly available due to privacy or ethical restrictions.

## Supplementary Materials

The following supporting information can be downloaded at https://www.mdpi.com/article/10.3390/jcm15062176/s1, Figure S1: Day-to-day evolution of Time-In-Bed across three phases (pre-treatment in dark grey, during treatment in light grey, and post-treatment in white) for both the stand-alone dCBT-I group (grey line) and the dCBT-I delivered within crenotherapy group (black line). Figure S2. Day-to-day evolution of Wake After Sleep Onset across three phases (pre-treatment in dark grey, during treatment in light grey, and post-treatment in white) for both the stand-alone dCBT-I group (grey line) and the dCBT-I delivered within crenotherapy group (black line). Figure S3. Day-to-day evolution of Total Sleep Time across three phases (pre-treatment in dark grey, during treatment in light grey, and post-treatment in white) for both the stand-alone dCBT-I group (grey line) and the dCBT-I delivered within crenotherapy group (black line). Figure S4. Day-to-day evolution of Sleep Onset Latency across three phases (pre-treatment in dark grey, during treatment in light grey, and post-treatment in white) for both the stand-alone dCBT-I group (grey line) and the dCBT-I delivered within crenotherapy group (black line). Figure S5. Day-to-day evolution of Late Insomnia across three phases (pre-treatment in dark grey, during treatment in light grey, and post-treatment in white) for both the stand-alone dCBT-I group (grey line) and the dCBT-I delivered within crenotherapy group (black line). Table S1. Sociodemographic, clinical and insomnia disorder characteristics at baseline in the stand-alone dCBT-I group, the dCBT-I delivered within a crenotherapy group, and the dropout and missing outcome data group.
