# Supplementary material for: Digital Cognitive Behavioural Therapy for Insomnia Delivered Within a Crenotherapy Setting: Results from a Multicentre Proof-of-Concept Randomised Controlled Trial
Source: J Clin Med. 2026 Mar 12;15(6):2176. doi: 10.3390/jcm15062176 (PMC13027175; doi:10.3390/jcm15062176)
Supplement: Supplementary file 1 [file jcm-15-02176-s001.zip › jcm-4169170-supplementary.pdf]

Supplementary Materials

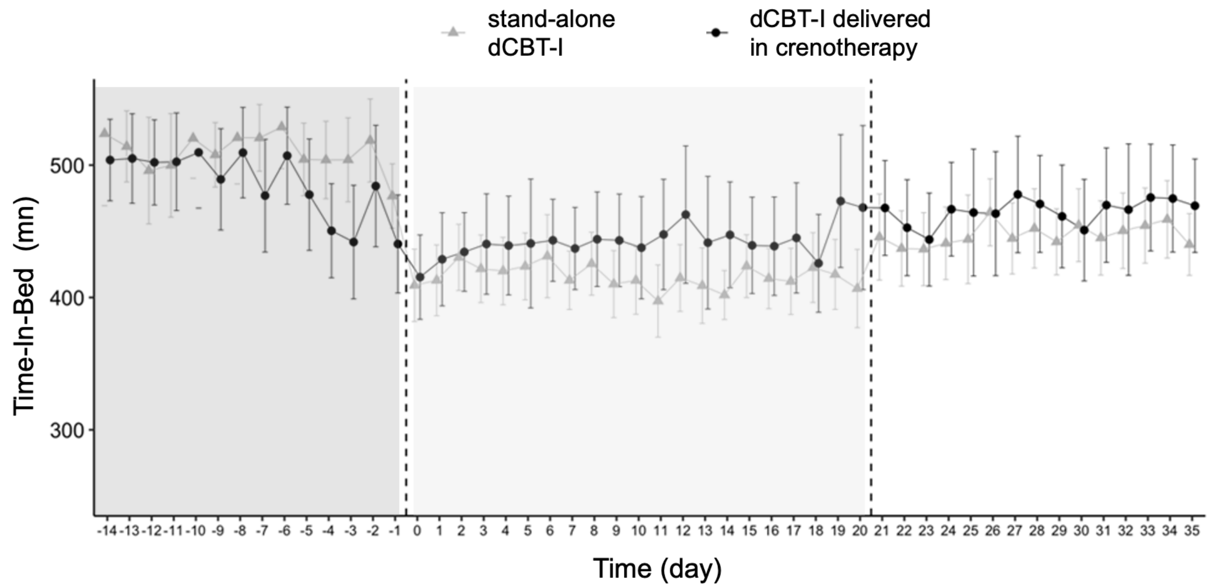

Figure 1. Day-to-day evolution of Time-In-Bed across three phases (pre-treatment in dark grey, during treatment in light grey, and post-treatment in white) for both the stand-alone dCBT-I group (grey line) and the dCBT-I delivered within crenotherapy group (black line).

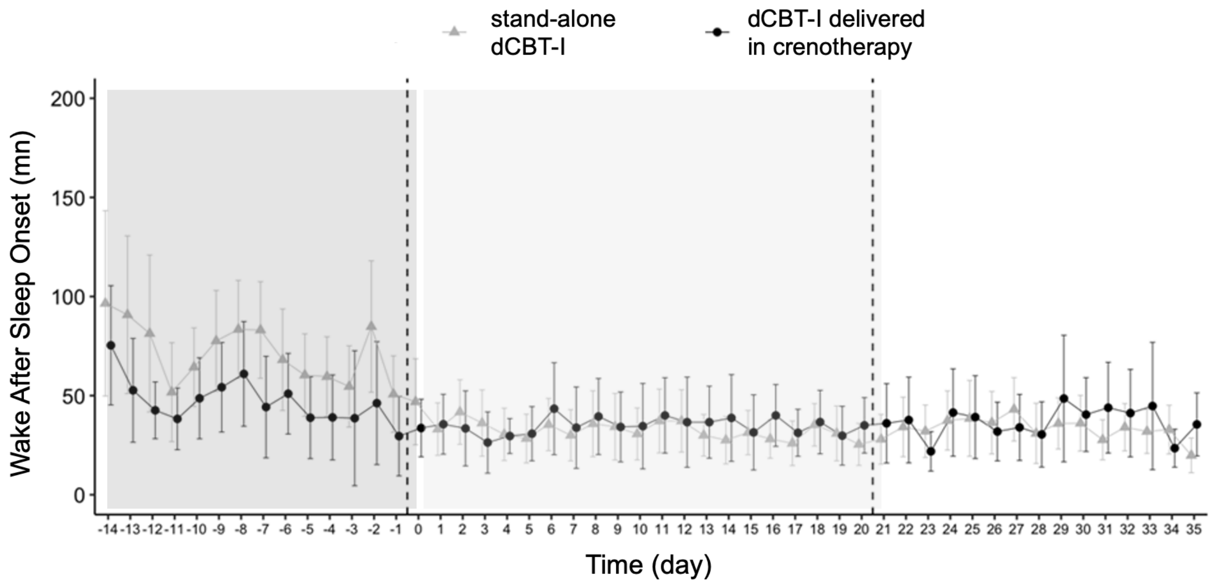

Figure 2. Day-to-day evolution of Wake After Sleep Onset across three phases (pre-treatment in dark grey, during treatment in light grey, and post-treatment in white) for both the stand-alone dCBT-I group (grey line) and the dCBT-I delivered within crenotherapy group (black line).

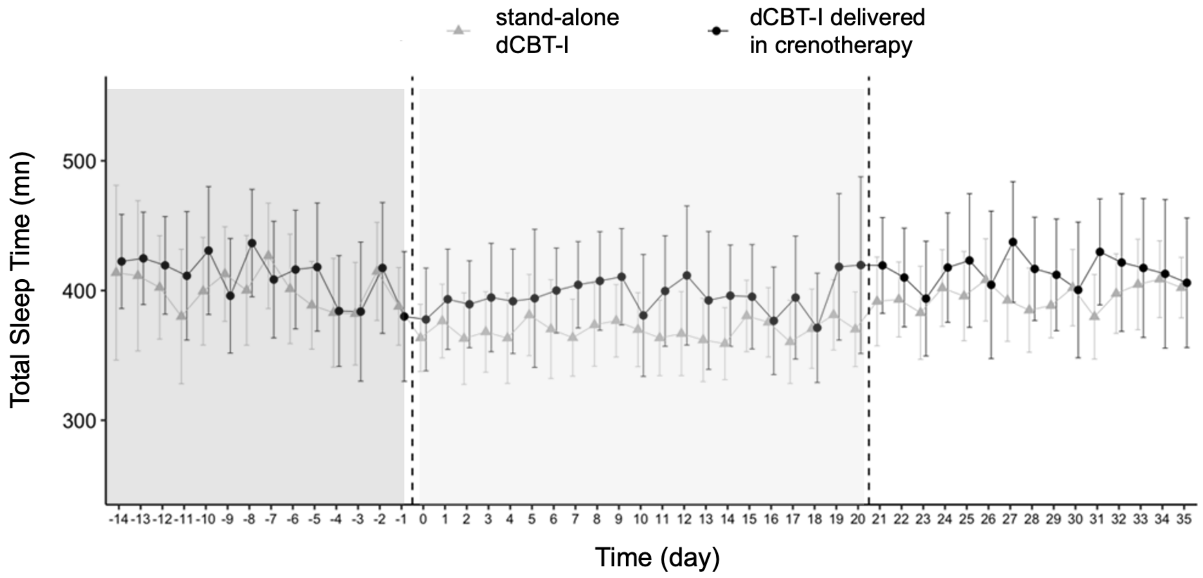

Figure 3. Day-to-day evolution of Total Sleep Time across three phases (pre-treatment in dark grey, during treatment in light grey, and post-treatment in white) for both the stand-alone dCBT-I group (grey line) and the dCBT-I delivered within crenotherapy group (black line).

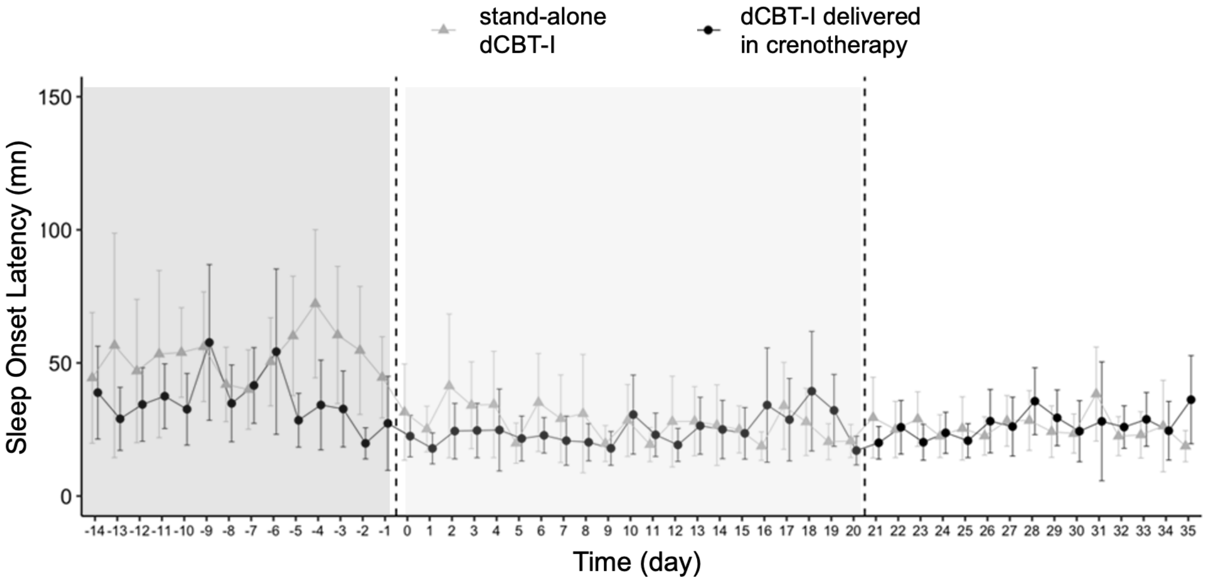

Figure 4. Day-to-day evolution of Sleep Onset Latency across three phases (pre-treatment in dark grey, during treatment in light grey, and post-treatment in white) for both the stand-alone dCBT-I group (grey line) and the dCBT-I delivered within crenotherapy group (black line).

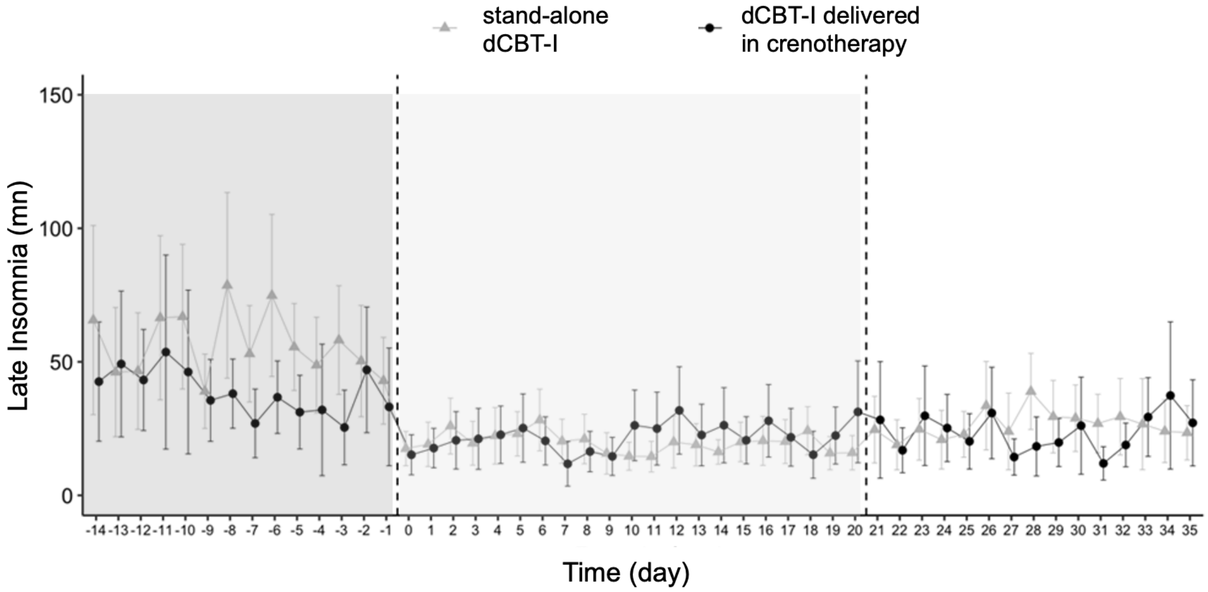

Figure 5. Day-to-day evolution of Late Insomnia across three phases (pre-treatment in dark grey, during treatment in light grey, and post-treatment in white) for both the stand-alone dCBT-I group (grey line) and the dCBT-I delivered within crenotherapy group (black line).

	Stand-alone dCBT-I Group and dCBT-I delivered in crenotherapy Group (*n* = 66)	Dropout and missing outcome data (*n* = 54)	*p* Value
Age, mean ± SD	59.18 ± 8.94	60.57 ± 11.38	0.459
Height, mean ± SD	164.82 ± 8.57	164.34 ± 7.32	0.747
Weight, mean ± SD	66.56 ± 14.93	67.21 ± 17.54	0.828
BMI, mean ± SD	24.43 ± 4.82	24.83 ± 6.06	0.689
Female, *n*, (%)	57 (86.36%)	46 (85.18%)	0.946
History of mental disorders, *n*, (%)	41 (62.12%)	26 (48.15%)	0.125
Previous crenotherapy, *n*, (%)	51 (77.27%)	46 (85.18%)	0.184
Hypnotic medication, *n*, (%)	36 (54.54%)	22 (40.74%)	0.132
ISI score, mean ± SD	19.77 ± 3.77	18.72 ± 3.74	0.130
Severe insomnia disorder (ISI> 21), *n*, (%)	22 (33.33%)	13 (24.07%)	0.295

Notes: BMI: Body Mass Index; ISI: Insomnia Severity Index; ISI>21 indicates severe insomnia disorder.

Table 1. Sociodemographic, clinical and insomnia disorder characteristics at baseline in the stand-alone dCBT-I group, the dCBT-I delivered within a crenotherapy group, and the dropout and missing outcome data group.
